# The mitogenomic landscape of *Banisteriopsis caapi* (Malpighiaceae), the sacred liana used for ayahuasca preparation

**DOI:** 10.1590/1678-4685-GMB-2023-0301

**Published:** 2024-07-01

**Authors:** Edisson Chavarro-Mesa, João Victor dos Anjos Almeida, Saura R. Silva, Simone Santos Lopes, Jose Beethoven Figueiredo Barbosa, Danilo Oliveira, Maria Alice Corrêa, Ana Paula Moraes, Vitor F.O. Miranda, Francisco Prosdocimi, Alessandro M. Varani

**Affiliations:** 1 Universidade Estadual Paulista “Júlio de Mesquita Filho” (UNESP), Faculdade de Ciências Agrárias e Veterinárias, Departamento de Biotecnologia Agropecuária e Ambiental, Jaboticabal, SP, Brazil.; 2 Universidade Estadual Paulista “Júlio de Mesquita Filho” (UNESP), Faculdade de Ciências Agrárias e Veterinárias, Departamento de Biologia, Jaboticabal, SP, Brazil.; 3 Universidade Estadual da Paraíba (UEPB), Laboratório de Genética e Biologia Molecular, Campina Grande, PB, Brazil.; 4 Universidade Federal de Roraima (UFRR), Departamento de Fitotecnia (DFIT), Boa Vista, RO, Brazil.; 5 Universidade Federal do Rio de Janeiro (UFRJ), Faculdade de Farmácia, Laboratório de Etnofarmacologia e Bioprospecção Aplicada, Rio de Janeiro, RJ, Brazil.; 6 Universidade Federal do Rio de Janeiro (UFRJ), Laboratório de Genômica e Biodiversidade, Instituto de Bioquímica Médica Leopoldo de Meis, Rio de Janeiro, RJ, Brazil.; 7 Universidade Federal do ABC (UFABC), Centro de Ciências Naturais e Humanas, São Bernardo do Campo, SP, Brazil.

**Keywords:** Entheogen, ethnobotany, conservation, comparative genomics, mariri/jagube

## Abstract

The sacred ayahuasca brew, utilized by indigenous communities in the Amazon and syncretic religious groups in Brazil, primarily consists of a decoction of two plants: (i) the Amazonian liana known as Mariri or Jagube (*Banisteriopsis caapi*), and (ii) the shrub referred as Chacrona or Rainha (*Psychotria viridis*). While Chacrona leaves are rich in N,N-Dimethyltryptamine (DMT), a potent psychedelic, the macerated vine of Mariri provides beta-carboline alkaloids acting as monoamine oxidase inhibitors, preventing DMT’s degradation. This study sequenced, assembled, and analyzed the complete genome of *B. caapi’*s mitochondrion, yielding a circular structure spanning 503,502 bp. Although the mtDNA encompasses most plant mitochondrial genes, it lacks some ribosomal genes, presents some atypical genes, and contains plastid pseudogenes, suggesting gene transfer between organelles. The presence of a 7-Kb repetitive segment containing copies of the rrnL and trnfM genes suggests mitogenome isomerization, supporting the hypothesis of dynamic mitogenome maintenance in plants. Phylogenetics and phylogenomics across 24 Malpighiales confirms the sample’s placement in the “Tucunacá” ethnovariety, aligning with morphological identification. This study spearheads efforts to decode the genome of this esteemed Malpighiaceae.

## Introduction

Nestled within the heart of the Amazon rainforest, *Banisteriopsis caapi* (Spruce ex Griseb.) C.V.Morton, commonly referred to as mariri, jagube, or yagé, is a liana deeply entrenched in indigenous lore. *B. caapi* is one of the 92 recognized species within the *Banisteriopsis* genus of the Malpighiaceae family (Gates, 1982). However, the monophyly of the *Banisteriopsis* genus remains a subject of controversy, as both morphological and molecular data reveal its members to be distributed among three distinct clades within the stigmaphylloids (Davis and Anderson, 2010). This family predominantly consists of diverse plant forms from subshrubs to trees (Gates, 1982; de Frias et al., 2012; Francener and Almeida, 2023). Beyond its botanical significance, when combined with leaves of Chacrona (*Psychotria viridis*), *B. caapi* becomes a key ingredient in ayahuasca, a ceremonial brew with deep spiritual connotations, often overseen by experienced regional shamans (Morales-García et al., 2017; dos Santos and Hallak, 2021). The traditional knowledge of indigenous people often describe mariri and chacrona as “teacher plants”, symbolizing their significance beyond mere botanical attributes.

Chacrona leaves contain significant amounts of N,N-Dimethyltryptamine (DMT), an analog of serotonin that acts as a powerful psychedelic and entheogen, being also produced in humans under certain specific circumstances, such as near-death experiences (Timmermann et al., 2018). When DMT is ingested, endogenous monoamine oxidase (MAO) enzymes encoded in the human genome often provide a quick degradation of this psychedelic molecule. Many species within the Malpighiaceae family produce secondary metabolites in the form of alkaloids that are capable of inhibiting the action of MAOs (Mannochio-Russo et al., 2022). The species sequenced here, *B. caapi,* is abundant in β-carboline alkaloids, including harmine, harmaline, and tetrahydroharmine, which act as potent monoamine oxidase inhibitors (MAOIs). In the ayahuasca, these MAOIs avoid the rapid degradation of DMT, allowing its effective transition to the bloodstream and subsequent impact on the brain synapses (Samoylenko et al., 2010; Morales-García et al., 2017; dos Santos and Hallak, 2021). These MAOIs, in addition to enhancing DMT’s effects, might also have inherent psychoactive properties, potentially modulating the intensity and duration of the ayahuasca experience (Riba et al., 2003).

*Banisteriopsis caapi* vine is noteworthy with several recognized ethnovarieties. Their primary phenotypic distinctions are evident in the stem morphology, specifically in the presence or absence of nodes (Luz et al., 2023). For example, two ethnovarieties frequently employed in ayahuasca preparation are (i) the Mariri “Tucunacá”, which lacks nodes, and (ii) the Mariri “Caupuri”, which often has them. However, numerous other ethnovarieties are utilized in ayahuasca preparations, not only by various Amazonian indigenous groups but also by syncretic religious organizations originating from northern Brazil (Luz *et al.*, 2023). Indeed, these diverse ethnovarieties may not only exhibit distinct phenotypic traits but also possess genomes that express unique MAOIs, leading to varied concentrations in plant tissues. Hence, the genomic exploration of Mariri has significance that reaches beyond conservation and ethnobotany, underscoring broader implications.

While the plastid genome sequence (ptDNA) of Mariri has been recently decoded (Ramachandran et al., 2018), the ultimate objective was to produce a resource that the U.S. Food and Drug Administration (FDA) could employ as target specific plant species using chloroplast genome sequences (Zhang et al., 2017). Consequently, only limited evolutionary insights and information about the components and structure of the *B. caapi* genome have been revealed.

In our current research, we sequenced and assembled the complete circular mitogenome (mtDNA) of a Mariri sample as an initial molecular resource of its whole genome that is currently under analysis. This sample was morphologically identified as the nodeless Tucunacá ethnovariety and sequenced using the PacBio Sequel II platform. Mitochondria are pivotal to eukaryotic cellular metabolism, responsible for oxidative phosphorylation and being the primary ATP source (Chandel, 2015; Møller et al., 2021). On the other hand, sequencing plant mtDNA is often challenging due to its repetitive nature, frequent rearrangements, and exogenous DNA presence (Mower et al., 2012; Arrieta-Montiel and Mackenzie, 2011; Møller *et al.*, 2021). Nonetheless, deciphering a plant mtDNA is paramount for our comprehension of plant evolution and diversity (Kan et al., 2020; Yu et al., 2023). As such, the mtDNA of *B. caapi* stands not only as the inaugural complete sequence for the Malpighiaceae family but also as a crucial genomic resource. This marks a significant stride towards a profound understanding of this honored plant.

## Material and Methods

### DNA extraction, sequencing and mitogenome assembly

*Banisteriopsis caapi* samples morphological identified as the nodeless Tucunacá ethnovariety were sourced from cultivation environment located at the União do Vegetal (UDV), Núcleo Menino Galante, situated in Serra da Cantareira, São Paulo, Brazil (coordinates: 23.380562 S, 46.58857 W). The voucher was cataloged at the Herbarium JABU at Universidade Estadual Paulista (Unesp), Jaboticabal campus (JABU1325).

The procedures involved in gathering, storing, DNA extraction, validating, sequencing and assembling the mtDNA were conducted following the same approach outlined in our previous study (Varani et al., 2022). In summary, one PacBio Sequel II SMRT cell was generated. Sequences from the GenBank Organelle Genome Resources repository (https://www.ncbi.nlm.nih.gov/genome/organelle/) were prepared for the Kraken2 database (Wood et al., 2019). Only reads specific to mtDNA were selected for assembly. The mtDNA assembly utilized a total of 152,628 HiFi reads (N50 of 18kb). Assembly was executed using Flye v2.9 (Kolmogorov et al., 2019), and the mtDNA molecule was extracted via “get_organelle_from_assembly.py” script from GetOrganelle (Jin et al., 2020). The resultant assembly graph was visualized with the Bandage software (Wick et al., 2015).

### Mitogenome annotation and visualization of the mitochondrial genome map

Annotation of the mitochondrial genome hinged on a combination of tools: GeSeq/Chlorobox (Tillich et al., 2017), MFannot (Lang et al., 2023), and Mitofy (Alverson et al., 2010). Each prediction was manually inspected to reach a consensus on gene annotation and to accurately pinpoint intron-exon boundaries and trans-splicing events. To achieve accurate gene annotation, we primarily relied on manually BLAST searches, using mitochondrial genes from reference and close-related species, allowing a putative inference of gene identity and function. For the determination of intron-exon boundaries, we carefully examined the predicted splice junction by manual inspection, supplemented by looking for conserved motifs and secondary structures that are characteristic of mitochondrial introns, especially in the case of trans-splicing events and comparative genomics data. Furthermore, for genes exhibiting trans-splicing, we used sequence alignment to identify and confirm the presence of separate gene fragments known to be joined post-transcriptionally. Overall, our consensus on gene annotation and the determination of intron-exon boundaries were reached through a combination of sequence similarity analysis using BLAST, careful examination of conserved genetic motifs and splice junction sequences. 

The previously published *B. caapi* ptDNA (Ramachandran et al., 2018) and the UniProt database (Apweiler et al., 2004), were used as reference for determination of the integration of plastid and other atypical genes. Only alignment hits showing >90% of coverage and >80% of identity, start and stop-codons were considered to determine a plastid or atypical gene as complete gene. Other predicted gene features, showing start and stop codons but with lower coverage (<60%) in comparison to the best hit homolog, were considered as incomplete genes. The mitochondrial genome map was made using both OGDraw (Greiner et al., 2019) and DNAPlotter (Carver et al., 2009). The mtDNA map’s aesthetics were fine-tuned and edited using Inkscape v1.3 (https://inkscape.org/release/inkscape-1.3/).

### Phylogenetic-based assessment of *Banisteriopsis caapi* ethnovariety and lineage

To ascertain the ethnovariety of our *Banisteropsis caapi* specimen, we retrieved sequences from the research of Luz et al. (2023) from the GenBank database. Specifically, 16 sequences corresponding to the ITS region (ITS-4 and ITS-5A; White, 1990) of the rDNA, (ON202484, ON202483, ON202482, ON202481, ON202476, ON202473, ON202474, ON202479, ON202475, ON202472, ON202477, ON202478, ON202480, ON202471, ON202470, ON202469) were selected. Our *B. caapi* ITS region was aligned using MAFFT v7.505 (default paramaters) with the corresponding sequences previously utilized to assess genetic diversity and identify ethnovarieties of *Banisteriopsis* spp. (Luz *et al.*, 2023). Phylogenetic reconstruction was undertaken using Bayesian analyses, leveraging the Markov chain Monte Carlo method alongside the Metropolis algorithm (MCMCMC) over 30,000,000 generations. The optimal evolutionary model, as defined by criteria such as the Symmetric model (SYM+G4), was chosen based on the AIC (Akaike, 1974) using ModelFinder (Kalyaanamoorthy et al., 2017), stationary trees have been removed and discarded the first 25% of trees generated. All analyses were executed with MrBayes v3.2.7a (Huelsenbeck and Ronquist, 2001). The resultant phylogenetic trees were visualized with Figtree v1.4.4 (http://tree.bio.ed.ac.uk/software/figtree).

### Phylogenomic analyses

Mitochondrial genomes from various Malpighiales species were retrieved from the GenBank database. Specifically, we used the genomes from Salicaceae (MW566588.1, NC_028096.1, NC_029317.1, NC_029693.1, NC_035157.1, NC_041085.1, NC_046754.1, NC_052708.1, NC_058733.1, NC_058734.1, NC_064688.1, NC_068760.1, NC_068761.1, NC_069586.1, ON064072.1), Passifloraceae (NC_050950.1), Calophyllaceae (OQ721861.1), Clusiaceae (OM759996.1), Rhizophoraceae (NC_056359.1, NC_069222.1), Euphorbiaceae (NC_015141.1, NC_045136.1, NC_056359.1, OQ658721.1, OQ658723.1), and, as outgroup, species of Brassicaceae (NC_037304.1, OQ852786.1), and Fabaceae (NC_048499.1, NC_048500.1, NC_048501.1) families. Next, 19 mtDNA genes (*atp1, atp4, atp6, atp8, ccmC, ccmFc, ccmFn, cob, cox1, cox2, cox3, matR, nad2, nad3, nad4, nad4L, rps12, sdh4*) were extracted, and aligned using MAFFT v7.505 with default parameters (Katoh and Standley, 2013). A concatenated matrix was generated using the catfasta2phyml script (https://github.com/nylander/catfasta2phyml). The optimal evolutionary model, as defined by criteria such as the General Time Reversible model (GTR+I+G4), was chosen based on the AIC (Akaike, 1974) using ModelFinder (Kalyaanamoorthy et al., 2017). Bayesian Markov chain Monte Carlo (MCMC) analyses were conducted with MrBayes v3.2.7a (Huelsenbeck and Ronquist, 2001), spanning thirty million generations, stationary trees have been removed and discarded the first 25% of trees generated. The resultant phylogenetic trees were visualized with Figtree v1.4.4 (http://tree.bio.ed.ac.uk/software/figtree).

### Nucleotide diversity assessment

For nucleotide diversity assessment the mitogenomes clustering with *B. caapi* in the phylogenetic tree, *Manihot esculenta* (NC_045136.1)*, Hevea pauciflora* (OQ_658723.1)*, Bruguiera sexangula* (NC_056359.1), and the external control *Arabidopsis thaliana* (NC_037304.1) were employed in the analysis. Using DNASP v4.5, we calculated the following metrics, such as polymorphic sites, number of informative parsimonious sites, number of unique variable sites, % G+C, % pairwise identical, % identical sites, and nucleotide diversity (Rozas and Rozas, 1999). 

### Comparative analysis

The average nucleotide identities among selected genomes were calculated using the “*ani.rb*” tool (https://github.com/lmrodriguezr/enveomics/blob/master/Scripts/ani.rb). The mtDNA sequences of *B. caapi*, *B. sexangula* (NC_056359.1), *H. pauciflora* (OQ_658723.1) and *M. esculenta* (NC_045136.1) were aligned using Sibelia software (Synteny Block ExpLoration tool) (Minkin et al., 2013). The results, encompassing visualizations of synteny and repeated segments, were visualized using Circos v0.69-6 (Krzywinski et al., 2009).

### Mitogenomes data availability

The *B. caapi* samples were recorded in the Brazilian National System of Management of Genetic Heritage and Associated Traditional Knowledge (SisGen) under access #A2A72C6. The *B. caapi* mtDNA genome and raw reads were deposited into GenBank, BioProject PRJNA997335 (mitogenome accession: OR473419, SRA raw reads accession: SRR25493516). 

## Results

### *Banisteriopsis caapi* mitogenome gene content and landmarks

**Figure 1 -:**
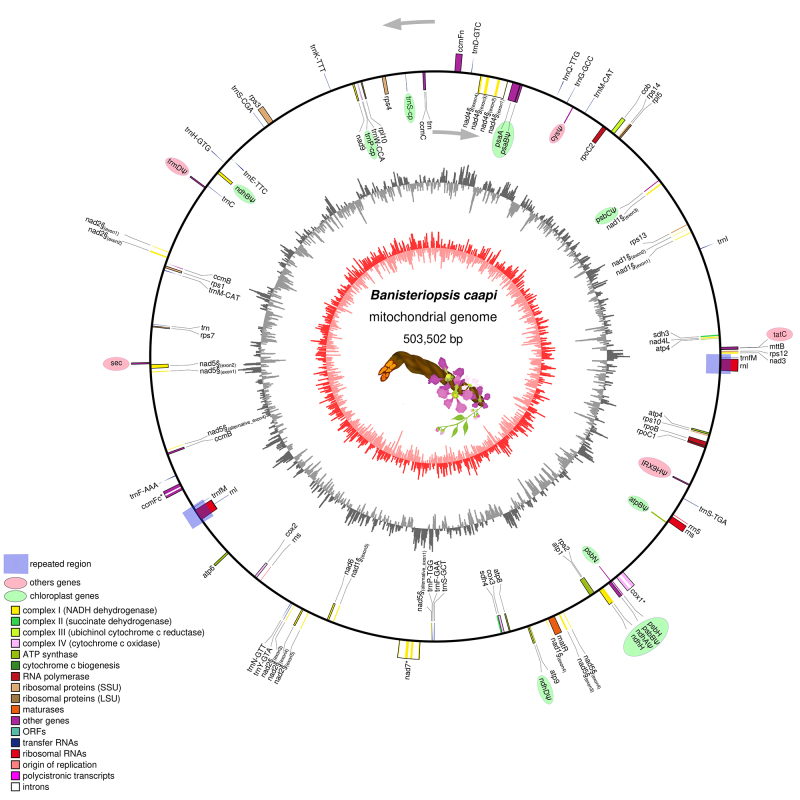
Circular representation of the *Banisteriopsis caapi* mtDNA. The circular genome is presented with genes transcribed clockwise on the outer circle and those transcribed counter-clockwise on the inner circle. Function-specific color coding for genes is provided in the legend. The 7-kb repeated region is demarcated by blue boxes, while unique regions appear as blue rectangles. The innermost circle delineates the GC Skew (illustrated in shades of red), and the outermost circle indicates the GC% content (depicted in grayscale).

We successfully assembled and annotated the mitogenome of the sacred liana *Banisteriopsis caapi*. The assembled mitogenome is represented by a circular sequence of 503,502 bp in length and possesses a GC content of 44.01% (Figure 1). A total of 59 genes were identified within the mitogenome: 36 protein-coding genes, 20 tRNA, and the rRNA operon (*rrnL*, *rrnS* and *rrn5*) (Table 1). As a main landmark, it was identified as a 7 Kb repeated region containing *rrnL* and *trnfM* genes copies. This repeated region may isomerize the mtDNA into two distinct sub-circles and isoforms by homologous recombination or other alternative recombination events mechanisms (Figure S1). Furthermore, while *nad1*, *nad2*, *nad4*, and *nad5* genes were predicted as subject to trans-splicing processes, the expected ribosomal genes *rps2*, *rps11*, *rpl2*, *rpl8*, *rpl11*, *rps19*, *rpl2*, *rpl6*, and *rpl16* were found to be absent. Despite these specifics, the size and arrangement of the mtDNA indicate that the mitogenome of *B. caapi* mirrors typical structures found in other plant mitochondrial genomes.

**Table 1 -:** Genes encoded by the *Banisteriopsis caapi* mtDNA.

Group of genes	Gene
Complex I (NADH dehydrogenase)	*nad1* ^*§[-]*^, nad2^*§[+]*^, nad3^*[+]*^, ad4^*§[-]*^, nad4L^*[-]*^, nad5^*§[-]*^ , nad6^*[-]*^, nad7^*[+]*^, nad9^*[-]*^
Complex II (succinate dehydrogenase)	*sdh3* ^*[-]*^, sdh4^*[-]*^
Complex III (ubiquinol cytochrome c reductase)	*cob* ^*[+]*^
Complex IV (cytochrome c oxidase)	*cox1* ^*[+]*^, cox2^*[+]*^, cox3^*[+]*^
Complex V (ATP synthase)	*atp1* ^*[-]*^, atp4^*[+]*^, atp6^*[+]*^, atp8^*[-]*^, atp9^*[+]*^
Cytochrome c biogenesis	*ccmB* ^*[-]+Ψ*^, ccmC^*[-]*^, ccmFc^*[+]*^, ccmFn^*[+]*^
Ribosomal proteins (SSU)	*rps1* ^*[-]*^, rps3^*[+]*^, rps4^*[-]*^, rps7^*[-]*^, rps10^*[-]*^, rps12^*[+]*^, rps13^*[-]*^, rps14^*[+]*^
Ribosomal proteins (LSU)	*rpl5* ^*[+]*^, rpl10^*[-]*^
Maturase	*matR* ^*[+]*^
Transport membrane proteins	*mttB* ^*[+]*^
tRNA and rRNA	*rrnl* ^*[+][x2]*^, rrnS^*[+][x2]*^, rrn5^*[+]*^, trnC (GCA)^*[-]*^, trnD (GTC)^*[+]*^, trnE (TTC)^*[-]*^, trnF (GAA)^*[-]*^, trnF (AAA)^*[+]*^, trnfM^*[-,+][2x]*^, trnG (GCC)^*[+]*^, trnH (GTG)^*[+]*^, trnI (Ile)^*[+]*^, trnK (TTT)^*[+]*^, trnM (CAT)^*[-][2x]*^, trnN (GTT)^*[+]*^, trnP (TGG)^*[-]*^, trnQ (TTG)^*[+]*^, trnS (CGA)^*[-]*^, trnS (TGA)^*[-]*^, trnS (GCT)^*[-]*^, trnW (CCA)^*[-]*^, trnY (GTA)^*[+]*^, tRNA-Undet^*[-]*^
ptDNA genes	*atpB* ^*Ψ[-]*^, ndhA^*Ψ[+]*^, ndhB^*Ψ[-]*^, ndhD^*Ψ[+]*^, ndhH^*[+]*^, psaA^*[-]*^, psaB^*Ψ[-]*^, psbB^*Ψ[+]*^, psbC^*Ψ[-]*^, psbH^*[+]*^, psbN^*[-]*^, rpoB^*Ψ[-]*^, rpoC1^*Ψ[-]*^, rpoC2^*Ψ[-]*^, trnS-cp^*[-]*^, trnP-cp^*[-]*^
Other genes	*Sulfate adenylyltransferase (cys)* ^*Ψ[+]*^, probable beta-1,4-xylosyltransferase (IRX9H)^*Ψ[-]*^, tRNA (Guanine-N1-)-methyltransferase (trmD)^*Ψ[+]*^, Secreted protein (sec)^*[+]*^, Sec-independent protein translocase component TatC (tatC)^*[+]*^

Genes found in the mitochondrial genome for *B. caapi*. Genes marked with [x2] are duplicated. Plus, and minus represent the presence of the gene in the direct strand [+] or complementary-reverse strand [-].Ψ for pseudogene; § for trans-splicing genes.

A particularly striking observation was the presence of numerous plastid genes identified as pseudogenes, such as *atpB*, *ndhA*, *ndhB*, *ndhD*, *psaB*, *psbB*, *psbC*, *psbH*, *rpoB*, *rpoC1*, *rpoC2*, and *trnS*-cp. However, at least four plastid genes (*ndhH, psaA*, *psbH*, and *psbN*) were completely predicted inside the mitogenome. Additionally, other atypical genes that appears as incomplete were also identified, including a Sec-independent protein translocase component (*tatC*), Sulfate adenylyltransferase (*cys*), tRNA (Guanine-N1-)-methyltransferase (*trmD*), and a putative beta-1,4-xylosyltransferase (IRX9H) (Table S1). This finding supports the occurrence of endosymbiotic gene transfer between organelles. 

**Figure 2 -:**
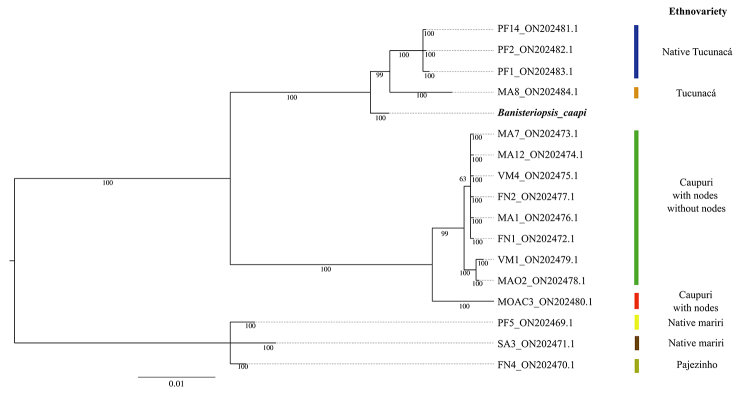
Phylogenetic reconstruction using the ITS marker performed through Bayesian analysis, elucidating the relationships among various ethnovarieties of *Banisteriopsis caapi*. Each taxon is identified by its corresponding ethnovariety, determined previously (Luz et al., 2023) and its associated GenBank accession number. The scale bar, located in the bottom-right corner, denotes the genetic distance measured in nucleotide substitutions.

**Figure 3 -:**
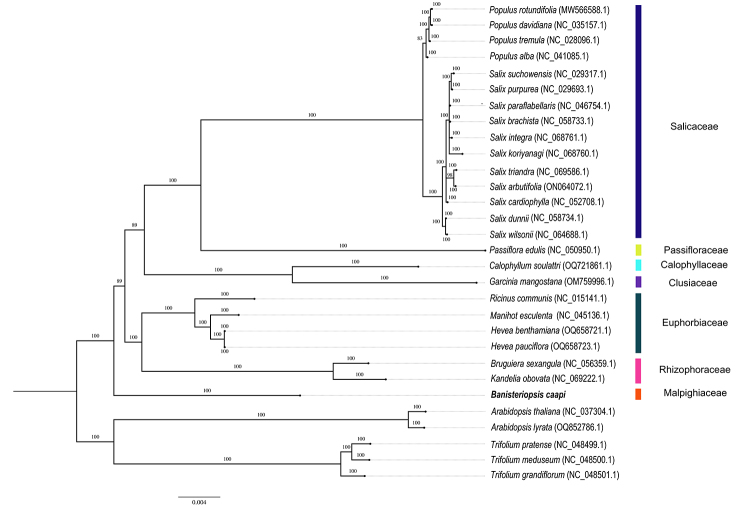
Bayesian tree of *Banisteriopsis caapi* mtDNA and related mitogenomes within the Malpighiales using all mtDNA CDS that share the 19 mtDNA genes. The *Arabidopsis thaliana* (NC_000932), *A. lyrata* (OQ852786.1), *Trifolium pratense* (NC_048499.1), *Trifolium meduseum* (NC_048500.1) and *Trifolium grandiflorum* (NC_048501.1) mtDNA were used as the outgroup. The scale bar value in the lower right corner represents the genetic distance in nucleotide substitutions.

The sample sequenced here clustered with others in the Tucunacá ethnovariety clade, confirming botanical identification (Figure 2). Furthermore, the phylogenomic analysis revealed that *B. caapi* occupies sister clade to other assessed Malpighiales order (Figure 3). In addition, our phylogenomic mitochondrial tree aligned Euphorbiaceae plants (*Ricinus communis*, *M. esculenta*, *Hevea benthamiana*, and *H. pauciflora*) with Rhizophoraceae species (*B. sexangula* and *Kandelia obovate)* in proximity to *B. caapi* of the Malpighiaceae family.

### Comparative analyses across other Malpighiales mitogenomes

**Figure 4 -:**
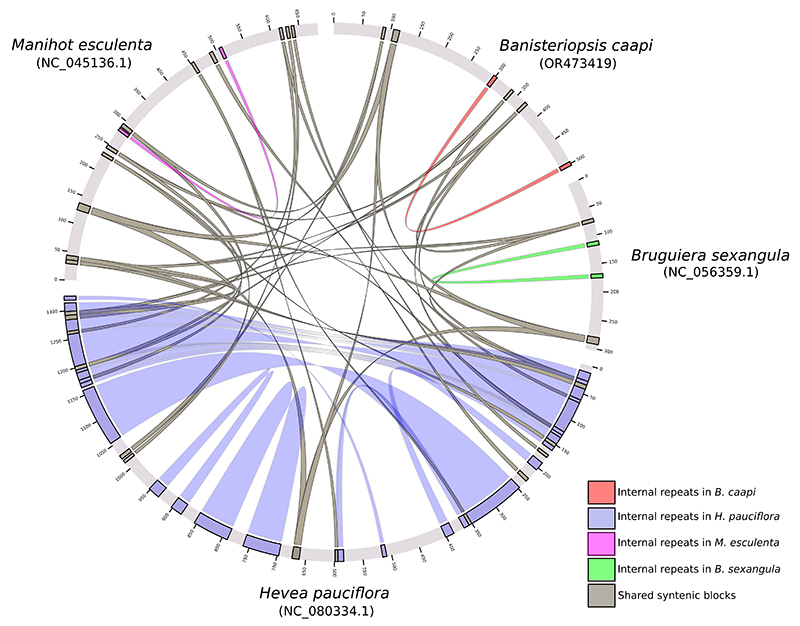
Comparative alignment of mitogenomes from *Banisteriopsis caapi*, *Hevea pauciflora*, *Manihot esculenta*, and *Bruguiera sexangula*. The shared and syntenic blocks are represented in gray, while the internal repeats are designated in light red (*B. caapi*), light blue (*H. pauciflora*), purple (*M. esculenta*), and light green (*B. sexangula*).

Further, we investigated the nucleotide diversity of the twenty-four mitochondrial genes shared among the species: *M. esculenta*, *H. pauciflora*, and *B. sexangula*. These analyses revealed an average of 42 polymorphic sites, 6 parsimonious sites, and 36 sites with unique variations. The nucleotide diversity was 0.03. The mean values for [G+C], pairwise identical, and identical sites were 42%, 96%, and 92%, respectively. Notably, the *sdh3* gene displayed the most significant nucleotide diversity, with a value of 0.19 (Table S2). At the macrosyntenic level, these mitogenomes display limited similarities, with the majority of syntenic blocks associated with gene regions (Figure 4). These observations are consistent with the species’ estimated divergence time and the typical plasticity observed in plant mitogenomes. Moreover, the Average Nucleotide Identity (ANI) for shared regions across these species approximates 93%.

## Discussion

In this study, we have sequenced, assembled and annotated the mtDNA of *B. caapi*, furnishing a resource for research in population genetics, genetic marker identification, and genetic diversity exploration among ethnovarieties. The analysis of this mitogenome helped in the understanding of *B. caapi* evolutionary path and its population interrelationships.

Also, the elucidation of its circular mitogenomic configuration, accompanied by an accurate enumeration of its mitochondrial genes, tRNAs, and rRNAs, provided a foundation for our ongoing research to unravel the complete genome sequence of this sacred species. Although *B. caapi* exhibits a typical mitogenome structure and composition, notable landmarks were revealed. For instance, the absence of specific genes like the ribosomal subunits *rps2*, *rps11*, *rpl2*, *rpl8*, *rpl11*, *rps19*, *rpl2*, *rpl6*, and *rpl16* suggests previous genomic rearrangements or deletions. Preliminary alignments of missing mitochondrial genes against a draft version of the complete genome of *B. caapi* (Prosdocimi and Varani *et al*., manuscript in preparation) returned “no hits”, suggesting that these genes were not transferred to the nuclear genome, being most likely deleted (data not shown). Also, mitochondria might be importing nuclear encoded ribosomes or ribosomal proteins. 

The detection of plastid genes in the mtDNA, alongside pseudogenization, highlights the interconnection between cellular compartments and potential mechanisms of genetic sharing among organelles, a feature commonly observed in mitochondrial genomes (Mower et al., 2012). On the other hand, it is crucial to consider that genes transferred from plastids might not function within the mitochondria, potentially undergoing pseudogenization as the mitochondrial genome evolves, thereby resulting in nonfunctional genes (Li et al., 2022).

The *B. caapi* also exhibited a 7 Kb repetitive region, containing copies of *rrnL* and *trnfM* genes, unveiling a complex genomic architecture that may be associated with previous genetic rearrangement events commonly observed in plant mitochondrial genomes and associated to multiple alternative arrangements (Kozik et al., 2019).

Moreover, the presence of atypical genes is particularly intriguing, because these genes do not seem to be directly connected to mitochondria biology. Despite these genes appearing as incomplete, they present start and stop codons, indicating that they may be transcribed and even translated as truncated proteins of unknown function. For instance, the *tatC* is responsible for the transport of folded proteins across the plasma membrane in bacteria and the thylakoid membrane in chloroplasts of plants (Palmer and Stansfeld, 2020; Schäfer et al., 2020). As a theoretical possibility, it might be used for the translocation of the missing ribosomal subunits across the mitochondrial membrane. Other atypical genes such as *cys* and IRX9H are described to be involved respectively in the assimilation of sulfate and synthesis of xyloglucans and other xylan-containing polysaccharides, which are major components of plant cell walls. This opens the possibility that the mitochondrial membrane of *B. caapi* might also be somehow glycosylated, potentially playing a role in regulating mitochondrial dynamics, biogenesis, and cellular stress responses. However, this hypothesis requires further research for substantiation and full understanding of these processes and their functional implications. The presence of *trmD* is intriguing as it catalyzes the methylation of guanine residues at the N1 position in certain tRNA molecules, stabilizing the structure of the tRNAs (Hou et al., 2017). Although the methylation machinery and many other components of the protein synthesis apparatus are encoded by nuclear genes and imported into the mitochondria, the *trmD* presence in the mtDNA may support the idea of its importance also for the protein synthesis at the mitochondrion*.* Indeed, debating the potential roles of non-standard genes in the mtDNA, their evolutionary origins, and conceivable cellular adaptations enriches our comprehension of metabolic pathways and transport processes within the plant mitochondrial framework and inspires future experimental work. Such revelations might herald a hitherto uncharted complexity in plant mitochondrial genomes. 

Also, understanding the genetic variations within *Banisteriopsis* genus is crucial for their effective conservation and a comprehensive ethnobotanical appreciation of these species. Luz et al. (2023) demonstrated the effectiveness of certain ptDNA genes and the ITS sequences as genetic barcodes for identifying and differentiating *Banisteriopsis* lineages. Employing these molecular markers, we were able to nest our sample with the “Tucunacá” ethnovariety lineage I, sourced from cultivation, and lineage II, derived from a natural environment (Luz *et al.*, 2023). This result also supports the morphological identification of our sample. Accurate identification facilitates the development of conservation strategies aimed at maintaining the genetic diversity within the species. Nonetheless, it is vital to emphasize that conserving this species should extend beyond the plants and natural environments, also encompassing the preservation of associated traditional knowledge systems and cultural practices.

Understanding the deep connections within Malpighiales is complicated due to the group’s ancient origins, which can be traced back to the mid-Cretaceous. This pattern is also observed in early-diverging angiosperms and Saxifragales. The complexity is further heightened by the uncertainty regarding their closest relatives, thereby complicating the comprehension of Malpighiales evolution (Wurdack and Davis, 2009). Indeed, our data adds valuable insights into the controversial phylogeny of Malpighiales species (Xi et al., 2012), a group that has been lacking sufficient mitochondrial genome data. With the sequencing of additional species, it is likely to provide further clarity and contribute significantly to unraveling the evolutionary history of this order. Our phylogenomic results further strengthened the evolutionary hypothesis sister clade to other assessed Malpighiales, one of first representatives that emerged at a basal position, which must be further confirmed with the sequencing and analysis of other mitogenomes. The phylogenomic results also spotlight the closest species with available genome sequences, paving the way for comparative genomic analysis in forthcoming efforts to generate chromosome-scale genome sequences for *B. caapi*.

Macrosyntenic analyses substantiate the idea that, despite discernible similarities among mitogenomes, their structural differences emphasize the dynamic nature of mtDNA maintenance. Notably, the mitochondrial genome showcases structural versatility, taking on forms ranging from a single expansive chromosome to numerous smaller ones in select species. However, its gene sequence remains notably conserved. Such observations imply that even if the structural layout of plant mitochondrial genomes may be mutable, their genetic encoded information is remarkably consistent (Møller et al., 2021), and this is not an exception for *B. caapi’s* mitogenome.

## Supplementary material

The following online material is available for this article:

Figure S1 - Repeats and putative recombinations in *Banisteriopsis caapi’s* mtDNA.

Table S1 - BLAST with plastid and other genes found in *Banisteriosis caapi’s* mtDNA.

Table S2 - Nucleotide diversity and neutrality tests for the mitogenomes sequences.

## References

[B1] Akaike H (1974). A new look at the statistical model identification. IEEE Trans Autom Control.

[B2] Alverson AJ, Wei X, Rice DW, Stern DB, Barry K, Palmer JD (2010). Insights into the evolution of mitochondrial genome size from complete sequences of Citrullus lanatus and Cucurbita pepo (Cucurbitaceae). Mol Biol Evol.

[B3] Apweiler R, Bairoch A, Wu CH (2004). Protein sequence databases. Current Opin Chem Biol.

[B4] Arrieta-Montiel MP, Mackenzie SA, Kempken F (2011). Plant mitochondria.

[B5] Carver T, Thomson N, Bleasby A, Berriman M, Parkhill J (2009). DNAPlotter: Circular and linear interactive genome visualization. Bioinformatics.

[B6] Chandel NS (2015). Evolution of mitochondria as signaling organelles. Cell Metabolism.

[B7] Davis CC, Anderson WR (2010). A complete generic phylogeny of Malpighiaceae inferred from nucleotide sequence data and morphology. Am J Bot.

[B8] de Frias UA, Costa MCM, Takahashi JA, Oki Y (2012). Banisteriopsis species: A source of bioactive of potential medical application. Int J Biotechnol Wellness Ind.

[B9] dos Santos RG, Hallak JEC (2021). Ayahuasca, an ancient substance with traditional and contemporary use in neuropsychiatry and neuroscience. Epilepsy Behav.

[B10] Gates B (1982). Banisteriopsis, Diplopterys (Malpighiaceae).

[B11] Greiner S, Lehwark P, Bock R (2019). OrganellarGenomeDRAW (OGDRAW) version 1.3.1: Expanded toolkit for the graphical visualization of organellar genomes. Nucleic Acids Res.

[B12] Hou YM, Matsubara R, Takase R, Masuda I, Sulkowska JI (2017). TrmD: A methyl transferase for tRNA methylation with m1G37. Enzymes.

[B13] Huelsenbeck JP, Ronquist F (2001). MRBAYES: Bayesian inference of phylogenetic trees. Bioinformatics.

[B14] Jin J-J, Yu W-B, Yang J-B, Song Y, dePamphilis CW, Yi T-S, Li D-Z (2020). GetOrganelle: A fast and versatile toolkit for accurate de novo assembly of organelle genomes. Genome Biol.

[B15] Kalyaanamoorthy S, Minh BQ, Wong TKF, von Haeseler A, Jermiin LS (2017). ModelFinder: Fast model selection for accurate phylogenetic estimates. Nat Methods.

[B16] Kan SL, Shen TT, Gong P, Ran JH, Wang XQ (2020). The complete mitochondrial genome of Taxus cuspidata (Taxaceae): Eight protein-coding genes have transferred to the nuclear genome. BMC Evol Biol.

[B17] Katoh K, Standley DM (2013). MAFFT multiple sequence alignment software version 7: Improvements in performance and usability. Mol Biol Evol.

[B18] Kolmogorov M, Yuan J, Lin Y, Pevzner PA (2019). Assembly of long, error-prone reads using repeat graphs. Nat Biotechnol.

[B19] Kozik A, Rowan BA, Lavelle D, Berke L, Schranz ME, Michelmore RW, Christensen AC (2019). The alternative reality of plant mitochondrial DNA: One ring does not rule them all. PLoS Genet.

[B20] Krzywinski M, Schein J, Birol I, Connors J, Gascoyne R, Horsman D, Jones SJ, Marra MA (2009). Circos: An information aesthetic for comparative genomics. Genome Res.

[B21] Lang BF, Beck N, Prince S, Sarrasin M, Rioux P, Burger G (2023). Mitochondrial genome annotation with MFannot: A critical analysis of gene identification and gene model prediction. Front Plant Sci.

[B22] Li J, Li J, Ma Y, Kou L, Wei J, Wang W (2022). The complete mitochondrial genome of okra (Abelmoschus esculentus): Using Nanopore long reads to investigate gene transfer from chloroplast genomes and rearrangements of mitochondrial DNA molecules. BMC Genomics.

[B23] Luz TZ, Cunha-Machado AS, da Silva Batista J (2023). First DNA barcode efficiency assessment for an important ingredient in the Amazonian ayahuasca tea: Mariri/jagube, Banisteriopsis (Malpighiaceae). Genet Resour Crop Evol.

[B24] Mannochio-Russo H, de Almeida RF, Nunes WD, Bueno PC, Caraballo-Rodríguez AM, Bauermeister A, Bolzani VS (2022). Untargeted metabolomics sheds light on the diversity of major classes of secondary metabolites in the Malpighiaceae botanical family. Front Plant Sci.

[B25] Minkin I, Patel A, Kolmogorov M, Vyahhi N, Pham S, Darling A, Stoye J (2013). Algorithms in bioinformatics.

[B26] Møller IM, Rasmusson AG, Van Aken O (2021). Plant mitochondria-past, present and future. Plant J.

[B27] Morales-García JA, De la Fuente Revenga M, Alonso-Gil S, Rodríguez-Franco MI, Feilding A, Perez-Castillo A, Riba J (2017). The alkaloids of Banisteriopsis caapi, the plant source of the Amazonian hallucinogen Ayahuasca, stimulate adult neurogenesis in vitro. Sci Rep.

[B28] Mower JP, Sloan DB, Alverson AJ, Wendel J, Greilhuber J, Dolezel J, Leitch I (2012). Plant genome diversity.

[B29] Palmer T, Stansfeld PJ (2020). Targeting of proteins to the twin‐arginine translocation pathway. Mol Microbiol.

[B30] Ramachandran P, Zhang N, McLaughlin WB, Luo Y, Handy S, Duke JA, Vasquez R, Ottesen A (2018). Sequencing the vine of the soul: Full chloroplast genome sequence of Banisteriopsis caapi. Genome Announc.

[B31] Riba J, Valle M, Urbano G, Yritia M, Morte A, Barbanoj MJ (2003). Human pharmacology of ayahuasca: Subjective and cardiovascular effects, monoamine metabolite excretion, and pharmacokinetics. J Pharmacol Exp Ther.

[B32] Rozas J, Rozas R (1999). DnaSP version 3: An integrated program for molecular population genetics and molecular evolution analysis. Bioinformatics.

[B33] Samoylenko V, Rahman MM, Tekwani BL, Tripathi LM, Wang YH, Khan SI, Muhammad I (2010). Banisteriopsis caapi, a unique combination of MAO inhibitory and antioxidative constituents for the activities relevant to neurodegenerative disorders and Parkinson’s disease. J Ethnopharmacol.

[B34] Schäfer K, Künzler P, Schneider K, Klingl A, Eubel H, Carrie C (2020). The plant mitochondrial TAT pathway is essential for complex III biogenesis. Curr Biol.

[B35] Tillich M, Lehwark P, Pellizzer T, Ulbricht-Jones ES, Fischer A, Bock R, Hou YMS (2017). GeSeq - versatile and accurate annotation of organelle genomes. Nucleic Acids Res.

[B36] Timmermann C, Roseman L, Williams L, Erritzoe D, Martial C, Cassol H, Laureys S, Nutt D, Carhart-Harris R (2018). DMT models the near-death experience. Front Psychol.

[B37] Varani AM, Silva SR, Lopes S, Barbosa JBF, Oliveira D, Corrêa MA, Prosdocimi F (2022). The complete organellar genomes of the entheogenic plant Psychotria viridis (Rubiaceae), a main component of the ayahuasca brew. PeerJ.

[B38] White TJ, White TJ, Bruns TD, Lee SB, Taylor JW (1990). PCR protocols, a guide to methods and applications.

[B39] Wick RR, Schultz MB, Zobel J, Holt KE (2015). Bandage: Interactive visualization of de novo genome assemblies. Bioinformatics.

[B40] Wood DE, Lu J, Langmead B (2019). Improved metagenomic analysis with Kraken 2. Genome Biol.

[B41] Wurdack KJ, Davis CC (2009). Malpighiales phylogenetics: Gaining ground on one of the most recalcitrant clades in the angiosperm tree of life. Am J Bot.

[B42] Xi Z, Ruhfel BR, Schaefer H, Amorim AM, Sugumaran M, Wurdack KJ, Endress PK, Matthews ML, Stevens PF, Mathews S (2012). Phylogenomics and a posteriori data partitioning resolve the Cretaceous angiosperm radiation Malpighiales. Proc Natl Acad Sci U S A.

[B43] Yu R, Chen X, Long L, Jost M, Zhao R, Liu L, Jiao Y (2023). De novo assembly and comparative analyses of mitochondrial genomes in Piperales. Genome Biol Evol.

[B44] Zhang N, Ramachandran P, Wen J, Duke JA, Metzman H, McLaughlin W, Ottesen AR, Timme RE, Handy SM (2017). Development of a reference standard library of chloroplast genome sequences, GenomeTrakrCP. Planta Med.

