# Supplementary material for: The mitogenomic landscape of Banisteriopsis caapi (Malpighiaceae), the sacred liana used for ayahuasca preparation
Source: Genet Mol Biol. 2024 Jul 1;47(2):e20230301. doi: 10.1590/1678-4685-GMB-2023-0301 (PMC11234496; doi:10.1590/1678-4685-GMB-2023-0301)
Supplement: Figure S1 - [file 1415-4757-GMB-47-02-e20230301-s1.pdf]

Genetics and Molecular Biology

https://doi.org/10.1590/1678-4685-GMB-2023-0301

Supplementary Material to "The Mitogenomic Landscape of *Banisteriopsis caapi* (Malpighiaceae), the Sacred Liana used for Ayahuasca preparation"

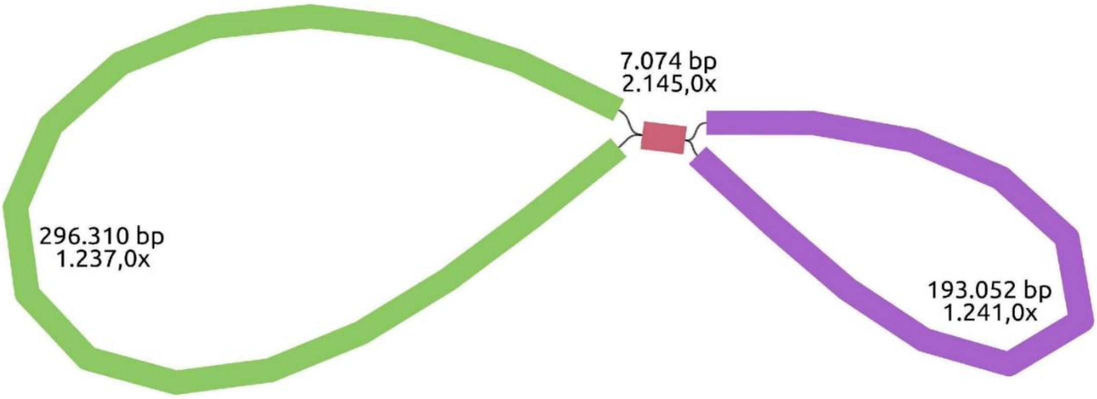

Figure S1 - Assembly graph visualization of Banisteriopsis caapi mtDNA reveals the repeats and possible sites for recombination that may generate other mtDNA isoforms.
