# Supplementary material for: The mitogenomic landscape of Banisteriopsis caapi (Malpighiaceae), the sacred liana used for ayahuasca preparation
Source: Genet Mol Biol. 2024 Jul 1;47(2):e20230301. doi: 10.1590/1678-4685-GMB-2023-0301 (PMC11234496; doi:10.1590/1678-4685-GMB-2023-0301)
Supplement: Table S1 - [file 1415-4757-GMB-47-02-e20230301-s2.pdf]

Genetics and Molecular Biology

https://doi.org/10.1590/1678-4685-GMB-2023-0301

Supplementary Material to "The Mitogenomic Landscape of *Banisteriopsis caapi* (Malpighiaceae), the Sacred Liana used for Ayahuasca preparation"

Table S1 - BLAST with chloroplast genes and other genes found in *Banisteriosis caapi* mtDNA.

Gene name	Coverage	E-value	Identity	Closest species	Accession
atpB	29%	0.0	96.11%	*Banisteriopsis caapi* plastid	NC_037945.1
ndhA	29%	5,00E-163	98.15%	*Banisteriopsis caapi* plastid	NC_037945.1
ndhB	50%	0.0	99.61%	*Banisteriopsis caapi* plastid	NC_037945.1
ndhD	31%	0.0	97.01%	*Banisteriopsis caapi* plastid	NC_037945.1
ndhH	92%	0.0	98.73%	*Banisteriopsis caapi* plastid	NC_037945.1
psaA	100%	0.0	99.82%	*Banisteriopsis caapi* plastid	NC_037945.1
psaB	27%	0.0	99.02%	*Banisteriopsis caapi* plastid	NC_037945.1
psbB	61%	0.0	99.15%	*Banisteriopsis caapi* plastid	NC_037945.1
psbC	10%	3,00E-52	89.86%	*Banisteriopsis caapi* plastid	NC_037945.1
psbH	100%	4,00E-113	98.20%	*Banisteriopsis caapi* plastid	NC_037945.1
psbN	100%	1,00E-28	90.70%	*Banisteriopsis caapi* plastid	NC_037945.1
rpoB	36%	0.0	98.01%	*Banisteriopsis caapi* plastid	NC_037945.1
rpoC1	21%	0.0	99.08%	*Banisteriopsis caapi* plastid	NC_037945.1
rpoC2	32%	0.0	96.78%	*Banisteriopsis caapi* plastid	NC_037945.1
tatC	86%	3,00E-160	89.96%	*Arabidopsis thaliana*	CAA51193.1
cys	15%	1,00E-44	83.75%	*Arabidopsis thaliana*	AAA92351.1
trmD	32%	4,00E-40	93.75%	*Striga asiatica*	GER38171.1
IRX9H	23%	5,00E-18	57.53%	*Arabidopsis thaliana*	AAD45998.1
sec	85%	7,00E-42	89.39%	*Digitaria exilis*	KAF8740720.1
