# Supplementary material for: The mitogenomic landscape of Banisteriopsis caapi (Malpighiaceae), the sacred liana used for ayahuasca preparation
Source: Genet Mol Biol. 2024 Jul 1;47(2):e20230301. doi: 10.1590/1678-4685-GMB-2023-0301 (PMC11234496; doi:10.1590/1678-4685-GMB-2023-0301)
Supplement: Table S2 - [file 1415-4757-GMB-47-02-e20230301-s3.pdf]

Genetics and Molecular Biology

https://doi.org/10.1590/1678-4685-GMB-2023-0301

Supplementary Material to "The Mitogenomic Landscape of *Banisteriopsis caapi* (Malpighiaceae), the Sacred Liana used for Ayahuasca preparation"

Table S2 - Nucleotide diversity and neutrality tests for the mitogenomes sequences shared among the species; *Banisteriopsis caapi*, NC_045136.1 *Manihot esculenta*, OQ_658723.1 *Hevea pauciflora*, and NC_056359.1 *Bruguiera sexangula*.

Gen	S	P	ns	π	% G+C	% Pairwise identical	% Identical sites	Sequence Length
atp1	66,0	20,0	46,0	0,02	44,10	97,50	95,50	1557
atp4	85,0	8,0	77,0	0,08	41,90	89,40	81,70	624
atp6	43,0	10,0	33,0	0,03	37,50	89,10	75,90	942
atp8	61,0	8,0	53,0	0,07	39,60	92,30	85,70	489
atp9	42,0	9,0	33,0	0,10	41,10	89,70	81,30	294
ccmB	38,0	3,0	35,0	0,03	41,00	96,20	92,90	621
ccmC	51,0	6,0	45,0	0,04	42,90	94,30	84,90	835
ccmFc	77,0	16,0	61,0	0,03	45,00	96,80	94,30	1347
ccmFn	96,0	7,0	89,0	0,03	46,20	95,30	90,80	1800
cob	28,0	3,0	25,0	0,01	41,40	98,60	97,40	1182
cox2	33,0	3,0	30,0	0,02	39,90	96,70	93,90	789
cox3	15,0	2,0	13,0	0,01	43,20	99,00	98,10	798
matR	84,0	14,0	70,0	0,02	51,40	97,20	94,70	1975
nad1	22,0	2,0	20,0	0,01	41,90	95,20	91,10	984
nad2	26,0	4,0	22,0	0,01	39,70	99,10	98,20	1467
nad3	10,0	1,0	9,0	0,01	39,80	98,60	97,20	357
nad4	29,0	10,0	19,0	0,01	40,70	98,90	98,10	1524
nad4L	6,0	0,0	6,0	0,01	35,80	97,00	94,10	303
nad5	55,0	10,0	45,0	0,01	40,40	97,90	96,20	2016
nad6	21,0	3,0	18,0	0,02	39,10	98,10	96,40	664
nad7	34,0	2,0	32,0	0,02	44,00	96,40	92,80	1185

Gen	S	P	ns	π	% G+C	% Pairwise identical	% Identical sites	Sequence Length
nad9i	12,0	2,0	10,0	0,01	41,40	98,90	97,90	573
rps12	11,0	1,0	10,0	0,01	44,00	98,50	97,10	378
sdh3	81,0	0,0	81,0	0,19	38,70	84,80	75,30	360
sdh4	18,0	4,0	14,0	0,03	37,90	96,70	94,00	426
**Average**	41,76	5,92	35,84	0,03	41,54	95,69	91,82	940

S = number of polymorphic (variable) sites; P = number of informative parsimonious sites; ns = number of unique variable sites; π (Pi) = nucleotide diversity.
